# Angiotensin II Receptor Blockers Improve Peripheral Endothelial Function: A Meta-Analysis of Randomized Controlled Trials

**DOI:** 10.1371/journal.pone.0090217

**Published:** 2014-03-03

**Authors:** Shuang Li, Yan Wu, Ge Yu, Qing Xia, Yawei Xu

**Affiliations:** 1 Department of Cardiology, Shanghai Tenth People's Hospital, Tongji University School of Medicine, Shanghai, China; 2 Department of Ophthalmology, Shanghai Tenth People's Hospital, Tongji University School of Medicine, Shanghai, China; 3 Department of Gastroenterology, Shanghai Tenth People's Hospital, Tongji University School of Medicine, Shanghai, China; Universtiy of Maryland School of Medicine, United States of America

## Abstract

**Objective(s):**

Several studies have assessed the effect of angiotensin II receptor blockers (ARBs) on peripheral endothelial dysfunction as measured by flow-mediated vasodilatation (FMD), a widely-used indicator for endothelial function. We conducted a meta-analysis to investigate the effect in comparison to placebo or no treatment and other antihypertensives.

**Methods:**

MEDLINE, Cochrane library and EMBASE were searched to September 2013 for randomized controlled trials (RCTs) that assessed the effect of ARBs versus placebo or no treatment and other antihypertensives (angiotensin-converting enzyme inhibitors (ACEIs), calcium channel blockers (CCBs), β-blockers, diuretics) by forearm FMD. Furthermore, we also use meta-regression to analyze the relationship between the endothelial function and the duration of ARBs treatments.

**Results:**

In 11 trials including 590 patients, ARBs (n = 315) significantly improved FMD (1.36%, 95% confidence internal [CI]:1.28 to 1.44) versus placebo or no treatment (n = 275). In 16 trials that included 1028 patients, ARBs (n = 486) had a significant effect (0.59%, 95% CI: 0.25 to 0.94) on FMD when compared with other antihypertensives (n = 542). In 8 trials, ARBs (n = 174) had no significant effect (−0.14%, 95% CI: −0.32 to 0.03) compared with ACEI (n = 173). Compared with others, the benefits of ARBs, respectively, were 1.67% (95% CI: 0.65 to 0.93) in 7 trials with CCBs, 0.79% (95% CI: 0.42 to 1.01) with β-blockers in 3 trials and 0.9% (95% CI: 0.77 to 1.03) with diuretics in 3 trials. Importantly, we found ARBs were less effective in a long time span (95% CI: −1.990 to −0.622) than the first 6 months (95% CI: −0.484 to 0.360).

**Conclusions:**

This study shows that ARBs improve peripheral endothelial function and are superior to CCBs, β-blockers and diuretics. However, the effect couldn't be maintained for a long time. In addition, there was no significant difference between ARBs and ACEI.

## Introduction

Endothelial dysfunction is an early marker for atherosclerosis and could be detected before structural changes to the vessel wall are apparent on angiography or ultrasound [1]. Several pathological conditions can lead to impairment of endothelial function, such as hypertension, diabetes, coronary artery disease and metabolic syndrome [2]. Examination of endothelium-dependent FMD using high-resolution ultrasonography is a widely-used noninvasive method of detecting endothelial dysfunction. It has also emerged that impaired FMD has a close correlation with the systemic nature of atherosclerosis and the future development and outcome of cardiovascular events [1], [2], [3].

The renin angiotensin system (RAS) plays a vital role in cardiovascular disease [1], [4], [5]. Angiotensin II receptor blockers (ARBs) inhibit the receptor of angiotensin II that stimulates the synthesis of nitric oxide (NO) and increases the levels of bradykinin to play a key role in vasodilatation and inhibition of vascular hypertrophy [5]. ARBs also promote an elastogenic profile in the extracellular matrix of the arterial wall by increasing elastin and decreasing the levels of matrix metalloproteinases. Similar mechanisms involved in regulating on RAS activity, ARBs and angiotensin-converting enzyme inhibitors (ACEIs) are both recommended first-line drugs for hypertension by guidelines [6], [7]. A prior meta-analysis [8] pooled that ACEIs could improve endothelial function in patients with endothelial dysfunction caused by various conditions. Whether ARBs are protective on endothelial function or superior to other antihypertensives reminds unclear.

Over the last decades, intensive research has investigated the potential clinical benefits of ARBs. Several clinical trials [9], [10], [11], [12], [13], [14], [15], [16], [17], [18], [19], [20], [21], [22], [23], [24], [25], [26], [27], [28], [29], [30] have tested the effect of ARBs on endothelial dysfunction using forearm FMD (brachial or radial artery) in patients with endothelial dysfunction caused by different pathological lesions. In this meta-analysis we investigated the ARBs compared with placebo or no treatment or other antihypertensives (ACEIs, CCBs, β-blockers, diuretics) on peripheral endothelial function as measured by FMD in patients with endothelial dysfunction.

## Materials and Methods

### Search strategy

Studies were eligible to be included in our meta-analysis if they were: (1) randomized controlled trials which compared any kinds of ARBs with monotherapy of placebo or no treatment or with other anti-hypertensives (ACEIs, CCBs, β-blockers or diuretics); (2) included patients with endothelial dysfunction (hypertension, type 2 diabetes, coronary artery disease, chronic kidney disease or elderly) as either the study population or a subgroup; (3) Used forearm FMD (Flow mediated vasodilatation or Flow mediated dilatation or Flow mediated dilation) measured by high-resolution ultrasound to assess peripheral endothelial function; (3) Minimum period of treatment with ARBs is more than 4 weeks or 1 month; (4) articles published in English until to September 2013.

### Data extraction

The following data were recorded for each study: first author, year of publication, country of research, number of participants randomized to ARBs and controls (not the total number that participated in the RCTs), age and gender, number of participants randomized into ARBs, placebo or no treatment and other antihypertensives respectively, ARBs type and dose, duration of treatment, controls type and dose, FMD at baseline and at the end of the study period, outcome. Authors of included studies were contacted when data was not available as appropriate (2 crossover trials as missing baseline FMD and 1 parallel double-blind trial as missing FMD change value). 2 independent reviewers (Shuang Li and Yan Wu) extracted and checked the data separately. Disagreements were resolved by consensus with the third reviewer, prof. Yawei Xu.

### Assessment of methodological quality

Two reviewers (Shuang Li and Yan Wu) assessed the methodological quality assessment independently and any incongruity was discussed and resolved. The methodological quality of the included studies was assessed by the elements of the Cochrane collaboration tool, by which the risk of bias in each trial was assessed [19]. A total of 7 domains were reported for each study including: random sequence generation, allocation concealment, blinding of participants, blinding of outcome assessment, follow-up, selective reporting and other bias. Jadad scoring system was adopted in the same time.

### Statistical methods for the meta-analysis

A random-effect model was obtained to conduct a meta-analysis of all the relevant RCTs to get a conservative conclusion. For continuous variables, weighted mean difference (WMD) was measured along with 95% CI, which is a measure of the likelihood of chance effects leading to random errors. A P value <0.05 was considered significant. The inter study heterogeneity was examined by both chi-squared test and I^2^ statistics. The heterogeneity was considered statistically significant when P<0.1 or I^2^>50%. Potential publication bias was assessed by both visual evaluation of funnel plot and Egger's test. The STATA (version 11.0; StataCorp) were used to conduct this meta-analysis. We also used meta-regression to test for a duration–effect relationship between duration of ARBs treatment and the change percent of FMD.

To explore the source of heterogeneity, each included study was removed one by one to detect its contribution on the heterogeneity. Besides, the meta-regression was also conducted to evaluate the source of heterogeneity. The sensitivity analysis was conducted excluding each study one at a time and then detecting the efficiency of ARBs on the FMD. The results would be considered robust when the results didn't change significantly.

## Results

### 1 Literature searching

**Figure 1:**
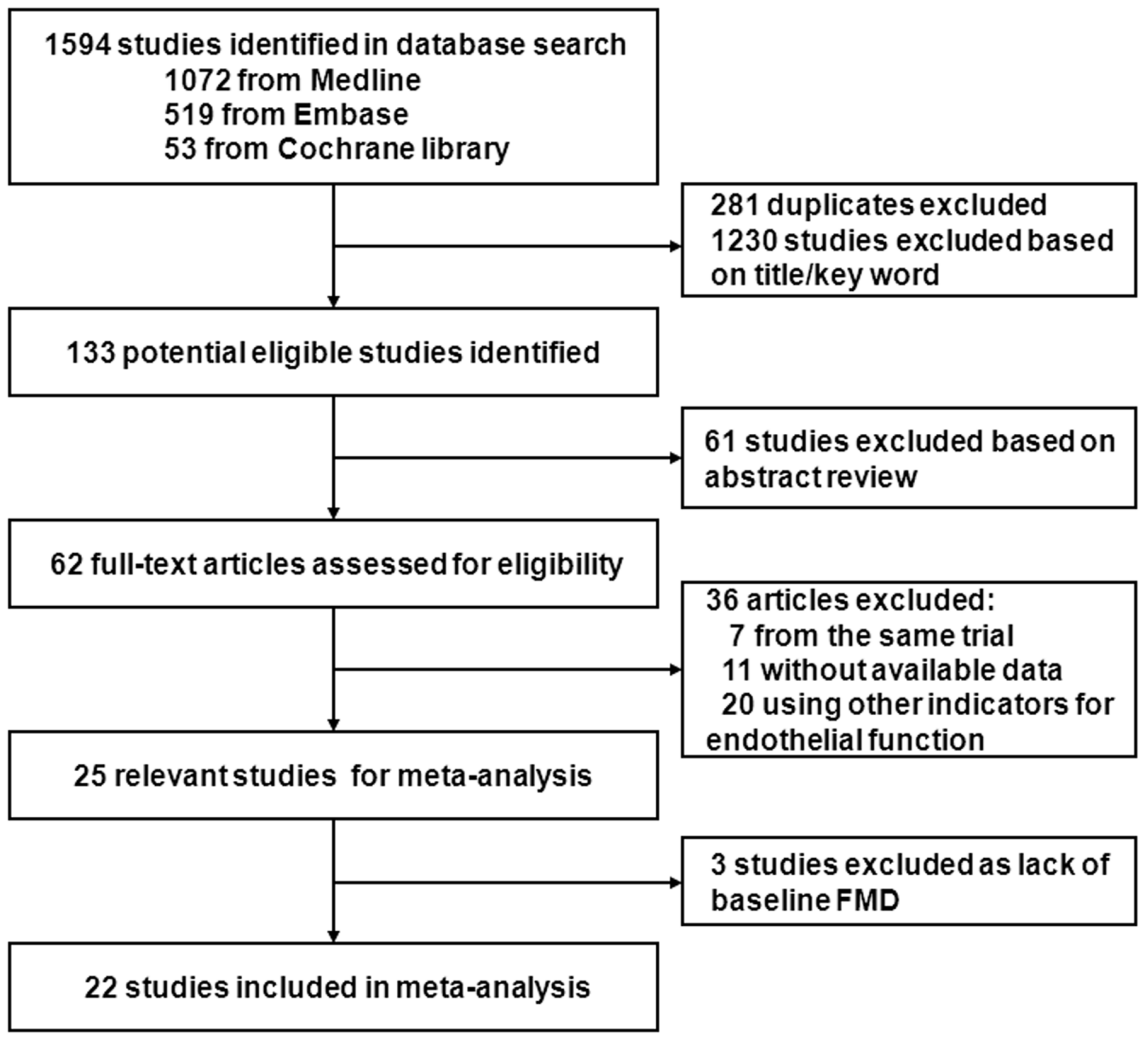
Study flow diagram and exclusion criteria. The searching protocol identified 1594 potentially eligible studies of which 281 were duplicated and 1230 studies were excluded on title and abstract. Full articles of the remaining 62 studies were collected and evaluated. 22 studies met our inclusion criteria and were included in the meta-analysis.

The searching protocol identified 1594 potentially eligible studies of which 281 were duplicated and 1230 studies were excluded on title and abstract. Full articles of the remaining 62 studies were collected and evaluated. 22 studies [9]–[30] met our inclusion criteria and were included in the meta-analysis.(see Fig.1) We classified included trials into two groups: 11 trials compared ARBs with placebo [9]–[16] or no treatment [17], [18], [19], 16 trials [15]–[30] compared ARBs with other antihypertensives [15], [17]–[30] (with ACEI in 8 trials [16]–[23], CCBs in 7 trials [15], [17], [24]–[28] β-blockers in 3 trials [15], [17], [29], diuretics in 3 trials [15], [19], [30]). 21 studies detected brachial FMD and 1 study detected radial FMD [20].

### 2 Characteristics of patients and trials

Our meta-analysis included 1737 patients of 11 countries and 22 trials totally (Table 1). In all trials, major characteristics of patients at baseline were similar between study groups. The mean age of patients ranged from 40 [21] to 71 [11] years old. 2 studies assessed ARBs effect on brachial FMD in male only [16], [18]. The mean systolic blood pressure ranged from 123 to 162.8 mmHg and the diastolic blood pressure ranged from 70 [11] to 100 mmHg [9], [17] at baseline. Mean follow-up duration ranged from 4 weeks [10], [14], [20], [29] to 3 years [23]. Patients' characteristics are summarized in Table 2.

**Table 1:** Characteristics of included trials.

First author, Year [Ref #]	Country	Protocol	Participants (n)	ARBs (n)	ARBs Dose/Day	Duration	Control (n)	Control Dose/Day	Outcome
***ARBs vs. placebo or no treatment***									
L.Ghiadoni,2003 [17]	Italy	R,P,SB	69	29	telmisartan,80–160 mg	6 m	40	no treatment	No effect on FMD
K.K.Koh,2004 [9]	Korea	R,DB,PC	122	92	Losartan,100 mg/irbesartan,300 mg/candesartan,16 mg	2 m	30	placebo	Improved FMD
J.Trevelyan,2005 [18]	UK	R,DB	33	18	losartan,50 mg	5 m	15	no treatment	Improved FMD
S.Sola,2005 [10]	USA	R,DB,PC	28	14	Irbesartan,150 mg	4 w	14	placebo	Improved FMD
L.A.Souza-Barbosa,2006 [19]	Brazil	R,O,P,PC	39	14	Irbesartan,150 mg	12 w	25	no treatment	Improved FMD
S.Rajagopalan,2006 [11]	USA	R,DB,PC,CO	33	33	Valsartan,160–320 mg	26 w	33	placebo	Improved FMD
A.Warnholtz,2007 [12]	Germany	R,DB,PC	63	30	Irbesartan,300 mg	6 m	33	placebo	Improved FMD
P.P.Filardi,2009 [13]	Italy	R,DB,PC	26	13	Candesartan,16 mg	2 m	13	placebo	Improved FMD
F.Pelliccia,2010 [14]	Italy	R,DB,P,PC	40	20	Telmisartan,160 mg	4 w	20	placebo	Improved FMD
K.K.Koh,2010 [15]	Korea	R,SB,PC,P	62	31	Candesartan,16 mg	8 w	31	placebo	Improved FMD
M.Lunder,2011 [16]	Slovenia	R,DB,PC	40	20	Valsartan,20 mg	30 d	20	placebo	Improved FMD
***ARBs vs. other antihypertensives (ACEI, CCB, β-blocker, diuretic)***									
***1. ARB vs. ACEI***									
B.Hornig,2001 [20]	Germany	R	35	17	losartan,100 mg	4 w	18	ramipril,10 mg	FMD improved under both treatments
L.Ghiadoni,2003 [17]	Italy	R,SB,P	57	29	telmisartan,160 mg	6 m	28	Perindopril,4 mg	FMD improved only under ACEI
D.Yavuz,2003 [21]	Turkey	R,O,P	18	9	Losartan,100 mg	6 m	9	Enalapril,40 mg	FMD improved under both treatments
J.Trevelyan,2005 [18]	UK	R,DB	34	18	losartan,50 mg	5 m	16	enalapril,10 mg	FMD improved under both treatments
L.A.Souza-Barbosa,2006 [19]	Brazil	R,O,PC	30	14	irbesartan,150 mg	12 w	16	Quinapril,20 mg	FMD improved under both treatments
K.K.Koh,2007 [22]	Korea	R,DB,CO,PC	34	34	candesartan,16 mg	4 m	34	ramipril,10 mg	FMD improved under both treatments
A.B.SOZEN,2009 [23]	Turkey	R	44	22	Irbesartan,300 mg/valsartan,160 mg	36 m	22	Fosinopril,10 mg/quinapril,20 mg	FMD improved under both treatments at the start of the trial but was not maintained
K.K.Koh,2010 [16]	Korea	R,SB,PC,P	61	31	Candesartan,16 mg	8 w	30	ramipril,10 mg	FMD improved under both treatments
***2. ARB vs. CCB***									
L.Ghiadoni,2003 [17]	Italy	R,P	85	29	Telmisartan,80–160 mg	6 m	56	nifedipine,30–60 mg/amlodipine,5–10 mg	No effect on FMD either.
S.Morimoto,2006 [24]	Japan	R	43	21	Telmisartan,40 mg	24 w	22	amlodipine,5 mg	ARB improved FMD than CCB
R.A.Benndorf,2007 [25]	Germany	R,SB,P	25	12	Telmisartan,40–80 mg	6 w	13	Nisoldipine,10–20 mg	ARB improved FMD than CCB
K.K.Koh,2010 [15]	Korea	R,SB,PC,P	61	31	Candesartan,16 mg	8 w	30	Amlodipine,10 mg	ARB improved FMD than CCB
M.I.Yilmaz,2010 [26]	Turkey	R	72	37	Valsartan,160 mg	12 w	35	amlodipine,10 mg	FMD improved under both treatments
D.Wei,2011 [27]	China	R,P,SB	55	27	olmesartan,20 mg	8 w	27	nisoldipine,10 mg	FMD improved under both treatments
S.Takiguchi,2011 [28]	Japan	R,CO	31	15	olmesartan,40 mg	12 w	16	amlodipine,10 mg	ARB improved FMD than CCB
***3. ARB vs. β-blocker***									
L.Ghiadoni,2003 [17]	Italy	R,P	86	29	telmisartan,80–160 mg	6 m	57	atenolol,50–100 mg/nebivolol,5–10 mg	No effect on FMD either.
A.J. Flammer,2007 [29]	Switzerland	R,DB,CO	14	14	losartan,100 mg	4 w	14	atenolol,100 mg	ARB improved FMD than β-blocker
K.K.Koh,2010 [15]	Korea	R,SB,PC,P	62	31	candesartan,16 mg	8 w	31	atenolol,100 mg	ARB improved FMD than β-blocker
***4. ARB vs. diuretics***									
N.A.Chung,2004 [30]	UK	R,DB,	40	21	losartan,50–100 mg	12 w	19	hydrochlorothiazide,12.5–25 mg	ARB improved FMD than diuretics
L.A.Souza-Barbosa,2006 [19]	Brazil	R,O,PC	32	14	irbesartan,150 mg	12 w	18	hydrochlorothiazide,20 mg	FMD improved under both treatments
K.K.Koh,2010 [15]	Korea	R,SB,PC,P	62	31	candesartan,16 mg	8 w	31	hydrochlorothiazide,50 mg	ARB improved FMD than diuretics

FMD flow mediated dilatation; O open; P parallel; CO crossover; PC placebo-control; DB double-blind; SB single-blind; R randomized; ARB angiotensin receptor blocker; ACEI angiotensin-converting enzyme inhibitors; CCB calcium channel blocker; NR not reported; m month; w week; d day.

**Table 2:** Characteristics of participants.

First author, Year(Ref #)	Participants	Mean Age	Male/Female	Mean SBP mmHg	Mean DBP mmHg	Smokers n (%)	Diabetes mellitus n%
***ARBs vs. placebo or no treatment***							
L.Ghiadoni,2003 [17]	essential hypertension;	49.8	42/27	132.4	87.1	NR	NR
K.K.Koh,2004 [9]	mild-to-moderate hypertension	48.5	96/26	162.8	100	0	0
J.Trevelyan,2005 [18]	stable CAD awaiting CABG	63.2	33/0	140	82	0	3(9.1)
S.Sola,2005 [10]	metabolic syndrome	41.5	12/16	133	77.5	NR	NR
L.A.Souza-Barbosa,2006 [19]	hypertension	47.9	17/22	138.9	83.5	NR	NR
S.Rajagopalan,2006 [11]	healthy normotensive elders	71	21/14	123	70	NR	NR
A.Warnholtz,2007 [12]	stable CAD	60	63/19	NR	NR	24(29.3)	11(13.4)
P.P.Filardi,2009 [13]	hypertension with stable CAD	58	27/1	123	78	25(89.2)	NR
F.Pelliccia,2010 [14]	normotensive patients with CAD	57	27/13	133	86	NR	9(22.5)
K.K.Koh,2010 [15]	hypertension	46.5	43/19	154	93.5	NR	0
M.Lunder,2011 [16]	healthy	43	40/0	123	75	0	0
***ARBs vs. other antihypertensives (ACEI, CCB, β-blocker, diuretic) 1. ARB vs. ACEI***							
B.Hornig,2001 [20]	CAD	59.5	NR	NR	NR	NR	NR
L.Ghiadoni,2003 [17]	hypertension	50.5	36/21	152	100	NR	NR
D.Yavuz,2003 [21]	hypertension	40	9/9	149	98	0	0
J.Trevelyan,2005 [18]	stable CAD awaiting CABG	63.8	34/0	143	81.6	2(5.9)	3(8.8)
L.A.Souza-Barbosa,2006 [19]	hypertension	49.5	13/17	158.4	92.1	NR	NR
K.K.Koh,2007 [22]	hypertension	46	23/11	155.5	95	11(32)	0
A.B.SOZEN,2009 [23]	mild-to-moderate hypertension	45	18/24	NR	NR	12(27.3)	20(45.5)
K.K.Koh,2010 [15]	hypertension	46.5	42/19	155	94	NR	NR
***2. ARB vs. CCB***							
L.Ghiadoni,2003 [17]	essential hypertension; normotensive subjects as control	51.6	52/33	152	100	NR	NR
S.Morimoto,2006 [21]	untreated hypertensive patients	57	18/25	162.5	94	11(25.6)	NR
R.A.Benndorf,2007 [25]	essential hypertension	57.9	13/12	NR	NR	1(4%)	NR
M.I.Yilmaz,2010 [26]	diabetic CKD stage I patients with hypertension	47	33/39	149	91	NR	72(100%)
K.K.Koh,2010 [15]	hypertension	49	41/20	155.5	95	NR	NR
D.Wei,2011 [27]	hypertension	58.6	41/14	148	87.8	NR	NR
S.Takiguchi,2011 [28]	essential hypertension	56	27/4	150.5	92.9	10(32.2)	7(22.6)
***3.ARB vs. β-blocker***							
L.Ghiadoni,2003 [17]	hypertension	52	53/33	153	99	NR	NR
A.J. Flammer,2007 [29]	type 2 diabetes and hypertension	61.3	10/3	133	82	6(46.2)	13(100)
K.K.Koh,2010 [15]	hypertension	48	43/19	156	95	NR	NR
***4.ARB vs. diuretics***							
K.K.Koh,2010 [15]	hypertension	47.5	42/20	154.5	94	NR	NR
L.A.Souza-Barbosa,2006 [19]	hypertension	49.8	13/19	156.8	91.1	NR	NR
N.A.Chung,2004 [30]	hypertension	55.9	28/12	161	95	10(25)	4(10)

### 3 The methodological quality of the included trials

The methodological quality of the included studies ranged from poor to moderate, with a median Jadad score of 3, range (1–3). This resulted from poor description of randomization and allocation concealment methods and the lack of double-blinding. Three studies [17], [19], [23] lacked adequate reporting on loss to follow up and withdrawals. In 2 studies [14], [30], diuretics were added to study treatments to normalize blood pressure in patients remaining hypertensive in spite of being on study treatments which might have affected the results too. Two studies [16], [18] included males only which might result in selective bias. Other aspects of methodological quality of the included trials are summarized in Table 3.

**Table 3:** Risk of bias assessment.

First author, Year(Ref #)	Adequate sequence generation	Allocation concealment	Blinding (observer)	Blinding (patient)	Adequate report on loss to follow-up	Free of other sourced of bias	Jadad score
***ARBs vs. placebo or no treatment***							
L.Ghiadoni,2003 [17]	Yes	NR	Yes	NO	NO	NO^a^	1
K.K.Koh,2004 [9]	Yes	NR	Yes	Yes	Yes	NO	3
J.Trevelyan,2005 [18]	Yes	NR	Yes	NO	Yes	NO^b^	3
S.Sola,2005 [10]	Yes	Yes	Yes	Yes	Yes	NO	3
L.A.Souza-Barbosa,2006 [17]	Yes	NO	NO	NO	NO	Yes	2
S.Rajagopalan,2006 [11]	Yes	NR	Yes	Yes	Yes	NO	3
A.Warnholtz,2007 [12]	Yes	NR	Yes	Yes	Yes	NO	3
P.P.Filardi,2009 [13]	Yes	NR	Yes	Yes	Yes	NO	3
F.Pelliccia,2010 [14]	Yes	NR	Yes	Yes	Yes	NO	3
K.K.Koh,2010 [15]	Yes	Yes	NO	Yes	Yes	Yes	3
M.Lunder,2011 [16]	Yes	NR	Yes	Yes	Yes	NO^b^	3
***ARBs vs. other antihypertensives(ACEI, CCB, β-blocker, diuretic)***							
B.Hornig,2001 [20]	Yes	NR	NO	NO	Yes	NO	1
D.Yavuz,2003 [21]	Yes	NO	NO	NO	Yes	Yes	2
K.K.Koh,2007 [22]	Yes	NR	Yes	Yes	Yes	Yes	3
A.B.SOZEN,2009 [23]	Yes	NO	NO	NO	NO	NO	1
S.Morimoto,2006 [24]	Yes	NO	NR	NR	NR	NO	1
R.A.Benndorf,2007 [25]	Yes	NR	NO	Yes	NR	NO	2
M.I.Yilmaz,2010 [26]	Yes	NR	NR	NR	NR	NO	1
D.Wei,2011 [27]	Yes	NR	NO	Yes	Yes	NO	2
S.Takiguchi,2011 [28]	Yes	NO	NO	NO	Yes	NO	1
A.J. Flammer,2007 [29]	Yes	NR	Yes	Yes	Yes	NO	3
N.A.Chung,2004 [30]	Yes	NR	Yes	Yes	Yes	NO^a^	3

a A diuretic was added to study treatments to normalize blood pressure.

b Included men only.

**NR** not reported; **ARB** Angiotensin receptor blocker; **ACEI** Angiotensin-converting enzyme inhibitors; **CCB** Calcium channel blocker.

### 4 FMD measurement

There was certain variation in the values of FMD among included studies. FMD change percent ranged from -0.3% [11] to 2% [19] in the placebo or no treatment group and 0.3% [17] to 5.9% [19] in relative ARBs groups. Also, when compared between ARBs with other antihypertensives, FMD ranged from −3.8% [23] to 5.6% [21] and −3.95% [23] to 5.9% [19] in relative ARBs groups. Other aspects of FMD measurements across included studies are summarized in Table 4.

**Table 4:** Technical aspects of forearm FMD measurement.

First author, Year(Ref #)	Mean change FMD (SD)		Probe (device) MHz	Position	Reproducibility
	ARB	Control			
***ARBs vs. placebo or no treatment***					
L.Ghiadoni,2003 [17]	0.3±2.9	0.1±1.06	NR	brachial artery	NR
K.K.Koh,2004 [9]	1.36±0.364	0.15±0.26	10(Bothell)	right brachial artery	NR
J.Trevelyan,2005 [18],2m	0.6±0.60	0.3±0.51	5-10(GE)	brachial artery	NR
J.Trevelyan,2005 [18],5m	3.3±0.65	2.0±0.68	5-10(GE)	brachial artery	NR
S.Sola,2005 [10]	2.7±0.82	0.2±0.98	NR	brachial artery	the average value was determined from at least 3 different measurements
L.A.Souza-Barbosa,2006 [19]	5.9±2.81	2±2.23	7-12(ALT HDI)	brachial artery	Mean difference between measures (0.9%); Intraobserver variability<2%
S.Rajagopalan,2006 [11]	0.8±0.9	−0.3±0.8	10(NR)	brachial artery	NR
A.Warnholtz,2007 [12]	2±0.65	0.3±0.56	NR	brachial artery	correlation coefficient (0.99)
P.P.Filardi,2009 [13]	1.88±2.3	1.39±2.15	7.5 (NR)	brachial artery	NR
F.Pelliccia,2010 [14]	4.9±3.5	0.6±3.2	7.5–12.5(Vivid 7)	right brachial artery	variability (0.38±0.26%);coefficient of variation(1.26%);coefficient of repeatability(0.5%) variability (0.01–0.02 mm)
K.K.Koh,2010 [15]	1.62±0.29	0.62±0.26	10(ATL)	right brachial artery	Interobserver variability (0.07–1.27%); Intraobserver variability (0.15–1.24%)
M.Lunder,2011 [16]	2.7±0.37	0.3±0.27	NR(Aloka alfa)	right brachial artery	NR
***ARBs vs. other antihypertensives (ACEI, CCB, β-blocker, diuretic) 1. ARB vs. ACEI***					
B.Hornig,2001 [20]	0.12±0.1	0.12±0.1	10(ASULAB)	radial artery	NR
L.Ghiadoni,2003 [17]	0.3±2.9	1.5±2.1	7(ESAOTE)	bradial artery	Intraobserver variability (14%); Mean difference between measures (0.9%)
D.Yavuz,2003 [21]	4.5±3.06	5.6±4.25	8.5(Logic 700)	bradial artery	Mean difference between measures (0.9%);Intraobserver variability (1–3%)
J.Trevelyan,2005 [18],2m	0.6±0.60	0.7±0.49	5-10(GE)	brachial artery	NR
J.Trevelyan,2005 [18],5m	3.3±0.65	3.9±0.56	5-10(GE)	brachial artery	NR
L.A.Souza-Barbosa,2006 [19]	5.9±2.81	6±2.48	7–12(ALT HDI)	bradial artery	Mean difference between measures (0.9%); Intraobserver variability<2%
K.K.Koh,2007 [22]	1.58±1.71	1.7±1.75	10(ATL)	brachial artery	NR
A.B.SOZEN,2009 [23]	−3.95±5	−3.8±4.6	10(VingMed)	brachial artery	Intra-andinter-observer variabilities 1–3%
K.K.Koh,2010 [15]	1.62±0.29	1.66±0.31	10(ATL)	right brachial artery	Interobserver variability (0.07–1.27%); Intraobserver variability (0.15–1.24%)
***2. ARB vs. CCB***					
L.Ghiadoni,2003 [17]	0.3±2.9	−0.4±2.45	7(ESAOTE)	brachial artery	Intraobserver variability (14%); Mean difference between measures (0.9%)
S.Morimoto,2006 [24]	3±0.92	−0.9±0.82	7.5(GE)	brachial artery	NR
R.A.Benndorf,2007 [25]	5.44±4.19	−0.68±3.57	12(ATL)	brachial artery	Mean intraindividual coefficient (4.2%)
K.K.Koh,2010 [15]	1.62±0.29	1.22±0.29	10(ATL)	right brachial artery	Interobserver variability (0.07–1.27%); Intraobserver variability (0.15–1.24%)
M.I.Yilmaz,2010 [26]	1.2±0.747	0.375±0.954	12(Bethell)	brachial artery	NR
D.Wei,2011 [27]	2.91±4.48	4±7.06	7.5(Philips)	brachial artery	The variability was 12% with a mean difference of 0.8% between the two measurements
S.Takiguchi,2011 [28]	1.59±2.92	0.04±2.34	7.5(Aplio)	right brachial artery	The intra- and inter-observer variability (<3%)
***3. ARB vs. β-blocker***					
L.Ghiadoni,2003 [17]	0.3±2.9	0.4±2.15	7(ESAOTE)	brachial artery	Intraobserver variability (14%) Mean difference between measures (0.9%)
A.J.Flammer,2007 [29]	0.73±0.43	−0.11±0.45	10(WTS-2)	brachial artery	NR
K.K.Koh,2010 [15]	1.62±0.29	0.8±0.35	10(ATL)	right brachial artery	Interobserver variability (0.07–1.27%); Intraobserver variability (0.15–1.24%)
***4. ARB vs. diuretics***					
N.A.Chung,2004 [30]	1.15±4.6	−0.26±4.571	10(GE)	brachial artery	NR
L.A.Souza-Barbosa,2006 [19]	5.9±2.81	5.5±2.722	7–12(ALT HDI)	brachial artery	Mean difference between measures (0.9%);Intraobserver variability<2%
K.K.Koh,2010 [15]	1.62±0.29	0.71±0.24	10(ATL)	right brachial artery	Interobserver variability (0.07–1.27%); Intraobserver variability (0.15–1.24%)

### 5 Outcome measures reporting

#### 5.1 ARBs versus placebo or no treatment

**Figure 2:**
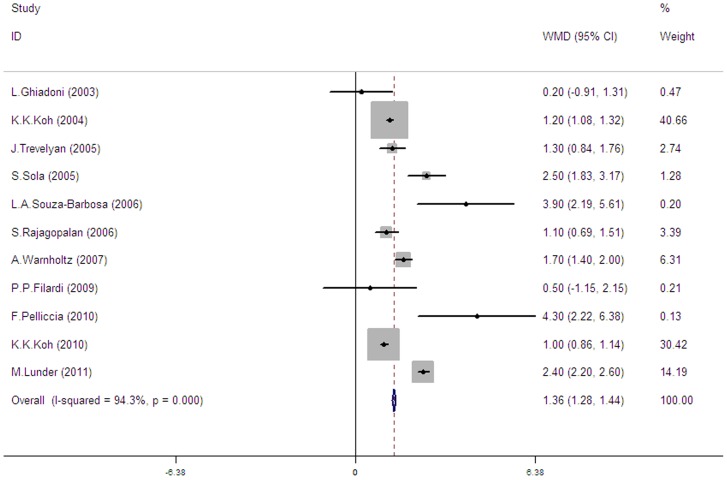
Forest plot illustrating ARBs effect on brachial FMD change compared with placebo or no treatment. In 11 trials including 590 patients, ARBs (n = 315) significantly improved FMD (1.36%, 95% CI: 1.28 to 1.44) versus placebo or no treatment (n = 275).

 Overall, 11 trials [9]–[19] assessed the effect of ARBs on FMD compared to placebo or no treatment, all using brachial arteries. These studies included 590 patients of which 315 patients received ARBs and 275 patients received placebo (8 trials [9]–[16]) or no treatment (3 trials [17], [18], [19]). Across the 11 trials we found significant heterogeneity (I^2^ = 94.3%, p<0.00001). A random effect model showed that treatment with ARBs significantly improved brachial FMD (pooled mean change difference  = 1.36%) (see Fig. 2).

#### 5.2 ARBs versus other antihypertensives

**Figure 3:**
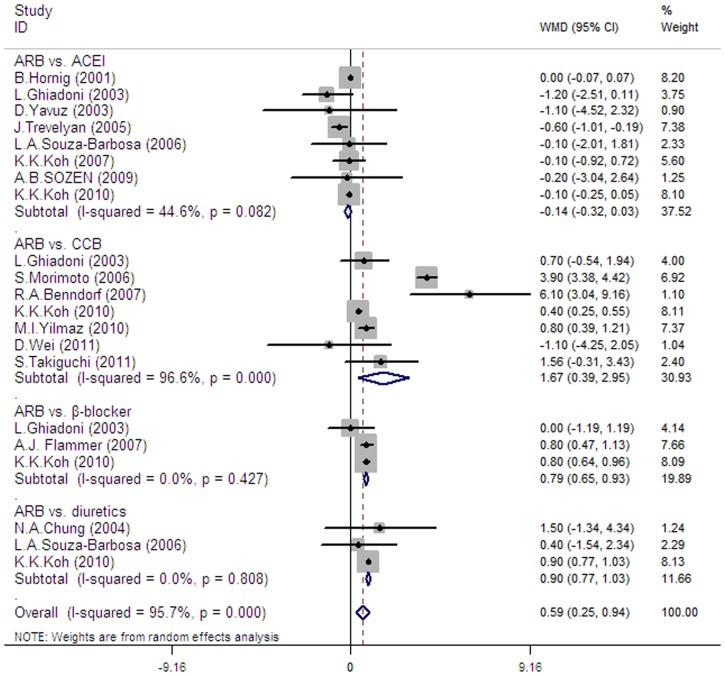
Forest plot illustrating ARBs effect on changes in FMD compared with other antihypertensive agents (ACEI, CCB, β-blockers and diuretics). In 16 trials that included 1028 patients, ARBs (n = 486) had a significant effect (0.59%, 95% CI: 0.25 to 0.94) on FMD when compared with other antihypertensives (n = 542). In 8 trials, ARBs (n = 174) had no significant effect (−0.14%, 95% CI: −0.32 to 0.03) compared with ACEI (n = 173). Compared with others, the benefits of ARBs respectively were 1.67% (95% CI: 0.65 to 0.93) in 7 trials with CCBs, 0.79% (95% CI: 0.42 to 1.01) with β-blockers in 3 trials and 0.9% (95% CI: 0.77 to 1.03) with diuretics in 3 trials.

In 15 trials (14 using brachial FMD and 1 using radial FMD [20]) which included 1028 patients, treatment with ARBs (n = 486) had a significant effect on FMD when compared with other antihypertensives (ACEI, CCBs, β-blockers and diuretics) (n = 542) (pooled mean change difference 0.59%, 95% CI 0.25–0.94, I^2^ = 95.7%, p for heterogeneity <0.00001) (see Fig. 3).

#### 5.3 ARBs versus ACEI

In 8 trials (7 with brachial FMD and 1 with radial FMD [20]), treatment with ARBs (n = 174) had no significant effect on FMD when compared with ACEI (n = 173) (pooled mean change difference  = −0.14%, 95% CI 0.32 to 0.03, p = 0.082, I^2^  = 37.52%).

#### 5.4 ARBs versus CCB

In 7 trials [15], [17], [24]–[28], all using brachial FMD, ARBs (n = 172) significantly improved FMD when compared with CCBs (n = 199) (pooled mean change difference 1.67%, 95% CI 0.65–0.93, I^2^ = 96.6%, p for heterogeneity <0.00001).

#### 5.5 ARBs versus β-blockers

When compared with β-blockers in 3 trials [15], [17], [29], all using brachial FMD, ARBs also had a significant effect on FMD (pooled mean change difference  = 0.79%, 95% CI 0.42–1.01, I^2^ = 0%, p for heterogeneity  = 0.427).

#### 5.6 ARBs versus diuretics

Three trials [15], [19], [30] which included 134 patients evaluated the effect of ARBs versus diuretics on FMD, all using brachial arteries, ARBs also had a significant effect on FMD (pooled mean change difference  = 0.9%, 95% CI 0.77–1.03, I^2^ = 0%, p for heterogeneity  =  0.808).

#### 5.7 Duration-effect relationship

**Figure 4:**
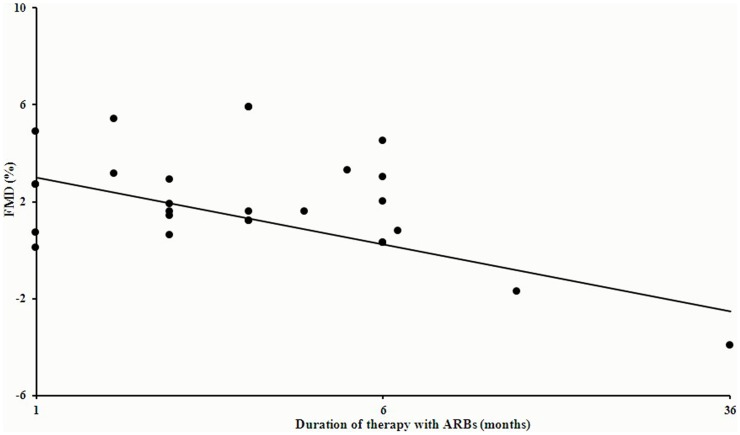
Relationship between the FMD change and the duration of ARBs treatments included all 22 trials. In available 25 values of total 22 studies, FMD change percent was relatively stable in the first 6 months (95% CI −0.484 to 0.360, p = 0.764), but felt down quickly after 6 month (95% CI−1.065 to 0.549, p = 0.154). In total, the benefit of ARBs on endothelial function wouldn't be well maintained (95% CI −1.990 to −0.622, p = 0.001).

We also analyze the relationship between the FMD change and the duration of ARBs treatments using meta-regression. In available 25 values of total 22 studies (J. Trevelyan [18] has 2 values of different time-points, A.B.SOZEN [23] has 3 values), of which 21 have results with follow-up less than or equal to 6 months and 2 trials [11], [23] contributed data more than 6 months. We found that the FMD change percent was relatively stable in the first 6 months (95% CI −0.484 to 0.360, p = 0.764) (see Fig.4), but felt down quickly after 6 month (95% CI-1.065 to 0.549, p = 0.154). In total, the benefit of ARBs on endothelial function wouldn't be well maintained (95% CI −1.990 to −0.622, p = 0.001) (see Fig. 4).

### 6 Publication bias

We found no significant evidence for publication bias. The Begg's test (P = 0.350) and the Egger's test (P = 0.357) also provided no statistical evidence for publication bias.

## Discussion

### Major findings

Our study is important in the sense that this is the first meta-analysis in the literature which assesses the effect of ARBs on peripheral endothelial function compared to placebo or no treatment and other antihypertensives. One of main findings of our meta-analysis is that treatments with ARBs could improve peripheral endothelial function compared with placebo or no treatment for patients who are suffering from endothelial dysfunction. When compared to other antihypertensives, ARBs show priority to CCBs, β-blockers and diuretics but have no significant difference with ACEI. Interestingly, although the anti-hypertensive targets of included RCTs have been well reached, we found the effect of ARBs on endothelial function still couldn't be well maintained in a long span. This suggested the improvement of endothelial function and reduction of blood pressure are not paralleled, which shined a deep clinical thinking that how to well set anti-hypertensive target based on the purpose of improvement of endothelial function.

### ARBs improve endothelial function

The mechanisms by which ARBs improve endothelial dysfunction are based on their ability of inhibiting angiotensin II receptor and to increasing bradykinin. On one hand, angiotensin II, by binding to angiotensin II type 1 receptor [5], leads to vascular constriction, endothelial cell migration, proliferation and hypertrophy and increases uptake and oxidation of LDL by endothelial cells as well as oxyradical production, thus leading to endothelial dysfunction [31]. On the other hand, bradykinin, via binding to the bradykinin B2 receptor, increases production and release of NO [32], prostacyclin [33] and the endothelium-derived hyperpolarizing factor [34] to cause vasodilatation, inhibition of vascular smooth muscle cell proliferation and platelet adhesion [35]. Therefore, inhibition of angiotensin II receptor and increasing bradykinin production by ARBs will result in an improvement in endothelial function.

Among the trials included in this study, variant members of this class (Table 1) that showed benefit on endothelial function are involved, including telmisartan (40 mg [24], 40–80 mg [25], 160 mg [14]), losartan (50 mg [18], 50–100 mg [30], 100 mg [9], [20], [21], [29]), irbesartan (150 mg [10], [19], 300 mg [9], [12], [23]), candesartan (16 mg [9], [13], [15], [22]) and valsartan (20 mg [16], 160 mg[23], [26], 160–320 mg [11]), thus to confirm that the protective effect on endothelial function is the common feature of ARBs

### ARBs versus ACEI

Recently, several studies have assessed the effect of ARBs and ACEIs on endothelial function as measured by brachial or radial FMD. As two antihypertensives in the involvement of renin angiotensin system (RAS), they have similar mechanisms. ACEIs could reduce production of angiotensin (Ang) II by inhibiting angiotensin converting enzyme, a key enzyme affecting the transformation from angiotensin I to angiotensin II [36]. Also, the inhibition of the angiotensin converting enzyme also increased bradykinin production [37]. However, the findings of“ACE escape” phenomenon [38] and other pathways, i.e. chymotrypsin-like enzymes that also induce Ang I to Ang II indicated ACE was not the major Ang II-forming enzyme [39], [40], thus directly given rise to the first member of ARBs, losartan. ARBs directly inhibited the binding between Ang II with its receptors, which seem more downstream and reliable. Consider the inhibition of Ang II is the leading mechanism of the improved endothelial function, ARB is seemed to superior to ACEIs. Since then, a wealth of clinical trial data has accumulated.

However, in this study, we pooled 8 studies that conducted ARBs and AECI monotherapy on endothelial dysfunction and found that 7 trials [15], [18], [19], [20], [21], [22], [23] show they can both improve FMD without significant difference but 1 trial [17] showed ARBs were less effective than ACEI. That's difficult to explain well using current mechanisms. Besides, some trials have compared the combination of ARBs and ACEI with monotherapy. K.K. Koh 2007 [22] demonstrated beneficial effects of the combination therapy superior to monotherapy with either drug and explained this by a greater extent of increased NO bioavailability. However, L.A. Souza-Barbosa [19] found that the combined effect of the two drugs on endothelial dysfunction was not better than either of these drugs separately. Hence, for this issue, we still need further evidence.

### FMD for endothelial function

For clinical assessment of endothelial function, FMD using high-resolution ultrasound to the forearm artery diameter is a well-known noninvasive method of detecting endothelial dysfunction. The mechanism is that reactive hyperemia induces increased blood flow and shear stress, stimulating NO release and vasodilation [1]. The systemic nature of atherosclerosis is reflected by the close correlation between endothelial dysfunction in the forearm and coronary endothelial dysfunction [41]. However, FMD to evaluate endothelial function also has some shortcomings. It is poor resolution relative to arterial size, highly operator-dependent and variable in measurements [3]. So it is important to note that in this meta-analysis we effect of ARBs on peripheral endothelial function base on FMD and there may be some certain variation existence.

### Duration-effect relationship between ARBs and endothelial function

A.B.SOZEN [23] indicated ARBs (irbesartan 300 mg/day or valsartan 160 mg/day) monotherapy improved endothelial function at 6 weeks after treatment, but this benefit fell below baseline at the one and three-year measurements, while there was no worsening in blood pressure control. Furthermore, our analysis all available data about the relationship between duration of ARBs therapy and FMD change percent and found that the FMD change percent was relatively stable within the first 6 months (95% CI −0.484 to 0.360, p = 0.764) (see Fig.4), but felt down quickly after 6 month (95% CI−1.065 to 0.549, p = 0.154). In total, the benefit of ARBs on endothelial function wouldn't be well maintained (95% CI −1.990 to −0.622, p = 0.001) (see Fig. 4), while BP targets were well reached in all trials. These results suggested that the improvement of endothelial function and lowering BP are not paralleled. Similar finding was also supported by Panza et al. [42], who found that endothelial impairment persisted after antihypertensive treatment had produced a clinical effect. Additionally, two studies [43], [44] suggested that very high doses of ARBs may exert a significantly greater anti-proteinuric action than standard doses, with no increment in the antihypertensive effect. We hypothesized that endothelial dysfunction might be resistant or irreversible if the pathological process is established and whether gradual increasing doses of ARBs with the extending of time of treatment may help to achieve persistent amelioration of endothelial function.

Endothelial function is known to be associated with the survival in patients with cardiovascular diseases and long-term use of ARBs is also associated with the survival in patients with cardiovascular diseases. However, we found that benefits of ARBs wouldn't provide persistent benefit on endothelial function in a long span. How to explain this paradox? On one hand, ARBs could not just improve endothelial dysfunction but also lower hypertension, relieve vascular remodeling and atherosclerosis as well as relieve metabolic syndrome et al [31]. On the other hand, this finding, benefits of ARBs on endothelial function couldn't be well maintained in a long span, was based on available data of 22 studies of which only 2 contributed follow-up data after 6 months [11], [23], thus need further more evidence for confirmation.

### Study strengths and limitations

Our study is important in the sense that this is the first meta-analysis of the literature which assessed the effect of ARBs on endothelial function as measured by fore-arm compared to placebo or no treatment and other antihypertensive agents. The strengths of this meta-analysis include a comprehensive literature search that included RCTs only. Although, authors of 3 trials (not included and cited) were contacted as missing baseline FMD value, we received no responses and therefore excluded them reluctantly. This may cause publication bias, although both tests by Begg's test (P = 0.350) and the Egger's test (P = 0.357) provided no statistical evidence. Another limitation of this meta-analysis is the high heterogeneity among included studies. This might be explained that patients included in our meta-analysis with endothelial dysfunction caused by different diseases.

Also, there are many widely-used methods to test endothelial function, including invasive methods, such as forearm blood flow, and non-invasive methods, such as FMD. In our study, we prefer to focus on the non-invasive methods and use FMD only for endothelial function, since it is more convenient for clinical application.

## Supporting Information

Checklist S1 **PRISMA checklist.**
(DOC)

## References

[1] Davignon J, Ganz P (2004) Role of endothelial dysfunction in atherosclerosis. Circulation 109: III27–32. 15198963 10.1161/01.CIR.0000131515.03336.f8

[2] Widlansky ME, Gokce N, Keaney JF Jr, Vita JA (2003) The clinical implications of endothelial dysfunction. J Am Coll Cardiol 42: 1149–1160. 14522472 10.1016/s0735-1097(03)00994-x

[3] Inaba Y, Chen JA, Bergmann SR (2010) Prediction of future cardiovascular outcomes by flow-mediated vasodilatation of brachial artery: a meta-analysis. Int J Cardiovasc Imaging 26: 631–640. 20339920 10.1007/s10554-010-9616-1

[4] Kawano H, Do YS, Kawano Y, Starnes V, Barr M, et al (2000) Angiotensin II has multiple profibrotic effects in human cardiac fibroblasts. Circulation 101: 1130–1137. 10715259 10.1161/01.cir.101.10.1130

[5] Watanabe T, Barker TA, Berk BC (2005) Angiotensin II and the endothelium - Diverse signals and effects. Hypertension 45: 163–169. 15630047 10.1161/01.HYP.0000153321.13792.b9

[6] Mancia G, Fagard R, Narkiewicz K, Redon J, Zanchetti A, et al. (2013) 2013 ESH/ESC Guidelines for the management of arterial hypertension. European Heart Journal 34: : 2159–+.

[7] James PA, Oparil S, Carter BL, Cushman WC, Dennison-Himmelfarb C, et al.. (2013) 2014 Evidence-Based Guideline for the Management of High Blood Pressure in Adults: Report From the Panel Members Appointed to the Eighth Joint National Committee (JNC 8). JAMA. 10.1001/jama.2013.284427 24352797

[8] Shahin Y, Khan JA, Samuel N, Chetter I (2011) Angiotensin converting enzyme inhibitors effect on endothelial dysfunction: a meta-analysis of randomised controlled trials. Atherosclerosis 216: 7–16. 21411098 10.1016/j.atherosclerosis.2011.02.044

[9] Koh KK, Han SH, Chung WJ, Ahn JY, Jin DK, et al (2004) Comparison of effects of Losartan, Irbesartan, and Candesartan on flow-mediated brachial artery dilation and on inflammatory and thrombolytic markers in patients with systemic hypertension. American Journal of Cardiology 93: 1432–1435. 15165934 10.1016/j.amjcard.2004.02.050

[10] Sola S, Mir MQS, Cheema FA, Khan-Merchant N, Menon RG, et al (2005) Irbesartan and lipoic acid improve endothelial function and reduce markers of inflammation in the metabolic syndrome - Results of the Irbesartan and Lipoic Acid in Endothelial Dysfunction (ISLAND) study. Circulation 111: 343–348. 15655130 10.1161/01.CIR.0000153272.48711.B9

[11] Rajagopalan S, Kariisa M, Dellegrottaglie S, Bard RL, Kehrer C, et al (2006) Angiotensin receptor blockade improves vascular compliance in healthy normotensive elderly individuals: results from a randomized double-blind placebo-controlled trial. J Clin Hypertens (Greenwich) 8: 783–790. 17086018 10.1111/j.1524-6175.2006.05797.x PMC8109297

[12] Warnholtz A, Ostad MA, Heitzer T, Thuneke F, Frohlich M, et al (2007) AT1-receptor blockade with irbesartan improves peripheral but not coronary endothelial dysfunction in patients with stable coronary artery disease. Atherosclerosis 194: 439–445. 16970950 10.1016/j.atherosclerosis.2006.08.034

[13] Perrone-Filardi P, Corrado L, Brevetti G, Silvestro A, Dellegrottaglie S, et al (2009) Effects of AT1 Receptor Antagonism With Candesartan on Endothelial Function in Patients With Hypertension and Coronary Artery Disease. Journal of Clinical Hypertension 11: 260–265. 19534023 10.1111/j.1751-7176.2009.00108.x PMC8673131

[14] Pelliccia F, Pasceri V, Cianfrocca C, Vitale C, Speciale G, et al (2010) Angiotensin II receptor antagonism with telmisartan increases number of endothelial progenitor cells in normotensive patients with coronary artery disease: A randomized, double-blind, placebo-controlled study. Atherosclerosis 210: 510–515. 20044087 10.1016/j.atherosclerosis.2009.12.005

[15] Koh KK, Quon MJ, Han SH, Lee Y, Kim SJ, et al (2010) Distinct vascular and metabolic effects of different classes of anti-hypertensive drugs. Int J Cardiol 140: 73–81. 19059660 10.1016/j.ijcard.2008.11.017 PMC2862263

[16] Lunder M, Janic M, Sabovic M (2012) Reduction of age-associated arterial wall changes by low-dose valsartan. Eur J Prev Cardiol 19: 1243–1249. 21933833 10.1177/1741826711423104

[17] Ghiadoni L, Magagna A, Versari D, Kardasz I, Huang Y, et al (2003) Different effect of antihypertensive drugs on conduit artery endothelial function. Hypertension 41: 1281–1286. 12719441 10.1161/01.HYP.0000070956.57418.22

[18] Trevelyan J, Needham EWA, Morris A, Mattu RK (2005) Comparison of the effect of enalapril and losartan in conjunction with surgical coronary revascularisation versus revascularisation alone on systemic endothelial function. Heart 91: 1053–1057. 16020596 10.1136/hrt.2004.036897 PMC1769026

[19] Souza-Barbosa LA, Ferreira-Melo SE, Ubaid-Girioli S, Arantes Nogueira E, Yugar-Toledo JC, et al. (2006) Endothelial vascular function in hypertensive patients after renin-angiotensin system blockade. J Clin Hypertens (Greenwich) 8: : 803–809; quiz 810–801. 17086021

[20] Hornig B, Landmesser U, Kohler C, Ahlersmann D, Spiekermann S, et al (2001) Comparative effect of ace inhibition and angiotensin II type 1 receptor antagonism on bioavailability of nitric oxide in patients with coronary artery disease: role of superoxide dismutase. Circulation 103: 799–805. 11171786 10.1161/01.cir.103.6.799

[21] Yavuz D, Koc M, Toprak A, Akpinar I, Velioglu A, et al (2003) Effects of ACE inhibition and AT1-receptor antagonism on endothelial function and insulin sensitivity in essential hypertensive patients. J Renin Angiotensin Aldosterone Syst 4: 197–203. 14608527 10.3317/jraas.2003.032

[22] Koh KK, Quon MJ, Lee Y, Han SH, Ahn JY, et al (2007) Additive beneficial cardiovascular and metabolic effects of combination therapy with ramipril and candesartan in hypertensive patients. Eur Heart J 28: 1440–1447. 17483542 10.1093/eurheartj/ehm101

[23] Sozen AB, Kayacan MS, Tansel T, Celebi A, Kudat H, et al (2009) Drugs with blocking effects on the renin-angiotensin-aldosterone system do not improve endothelial dysfunction long-term in hypertensive patients. J Int Med Res 37: 996–1002. 19761681 10.1177/147323000903700403

[24] Morimoto S, Yano Y, Maki K, Sawada K (2006) Renal and vascular protective effects of telmisartan in patients with essential hypertension. Hypertens Res 29: 567–572. 17137211 10.1291/hypres.29.567

[25] Benndorf RA, Appel D, Maas R, Schwedhelm E, Wenzel UO, et al (2007) Telmisartan improves endothelial function in patients with essential hypertension. J Cardiovasc Pharmacol 50: 367–371. 18049303 10.1097/FJC.0b013e31811dfbe7

[26] Yilmaz MI, Carrero JJ, Martin-Ventura JL, Sonmez A, Saglam M, et al (2010) Combined therapy with renin-angiotensin system and calcium channel blockers in type 2 diabetic hypertensive patients with proteinuria: effects on soluble TWEAK, PTX3, and flow-mediated dilation. Clin J Am Soc Nephrol 5: 1174–1181. 20430947 10.2215/CJN.01110210 PMC2893063

[27] Wei D, He WY, Lv QZ (2012) Effect of nisoldipine and olmesartan on endothelium-dependent vasodilation in essential hypertensive patients. CNS Neurosci Ther 18: 400–405. 22533725 10.1111/j.1755-5949.2012.00304.x PMC6493574

[28] Takiguchi S, Ayaori M, Uto-Kondo H, Iizuka M, Sasaki M, et al (2011) Olmesartan improves endothelial function in hypertensive patients: link with extracellular superoxide dismutase. Hypertens Res 34: 686–692. 21307868 10.1038/hr.2011.11

[29] Flammer AJ, Hermann F, Wiesli P, Schwegler B, Chenevard R, et al (2007) Effect of losartan, compared with atenolol, on endothelial function and oxidative stress in patients with type 2 diabetes and hypertension. J Hypertens 25: 785–791. 17351370 10.1097/HJH.0b013e3280287a72

[30] Chung NA, Beevers DG, Lip G (2004) Effects of losartan versus hydrochlorothiazide on indices of endothelial damage/dysfunction, angiogenesis and tissue factor in essential hypertension. Blood Press 13: 183–189. 15223728 10.1080/08037050410033312

[31] Bell L, Madri JA (1990) Influence of the angiotensin system on endothelial and smooth muscle cell migration. Am J Pathol 137: 7–12. 2164777 PMC1877705

[32] Barrow SE, Dollery CT, Heavey DJ, Hickling NE, Ritter JM, et al (1986) Effect of vasoactive peptides on prostacyclin synthesis in man. Br J Pharmacol 87: 243–247. 3513880 10.1111/j.1476-5381.1986.tb10177.x PMC1916907

[33] O'Kane KP, Webb DJ, Collier JG, Vallance PJ (1994) Local L-NG-monomethyl-arginine attenuates the vasodilator action of bradykinin in the human forearm. Br J Clin Pharmacol 38: 311–315. 7833219 10.1111/j.1365-2125.1994.tb04359.x PMC1364773

[34] Mombouli JV, Illiano S, Nagao T, Scott-Burden T, Vanhoutte PM (1992) Potentiation of endothelium-dependent relaxations to bradykinin by angiotensin I converting enzyme inhibitors in canine coronary artery involves both endothelium-derived relaxing and hyperpolarizing factors. Circ Res 71: 137–144. 1318793 10.1161/01.res.71.1.137

[35] Linz W, Wohlfart P, Scholkens BA, Malinski T, Wiemer G (1999) Interactions among ACE, kinins and NO. Cardiovasc Res 43: 549–561. 10690327 10.1016/s0008-6363(99)00091-7

[36] Alreja G, Joseph J (2011) Renin and cardiovascular disease: Worn-out path, or new direction. World J Cardiol 3: 72–83. 21499495 10.4330/wjc.v3.i3.72 PMC3077814

[37] Pellacani A, Brunner HR, Nussberger J (1994) Plasma kinins increase after angiotensin-converting enzyme inhibition in human subjects. Clin Sci (Lond) 87: 567–574. 7874846 10.1042/cs0870567

[38] Biollaz J, Brunner HR, Gavras I, Waeber B, Gavras H (1982) Antihypertensive therapy with MK 421: angiotensin II—renin relationships to evaluate efficacy of converting enzyme blockade. J Cardiovasc Pharmacol 4: 966–972. 6185790

[39] Urata H, Healy B, Stewart RW, Bumpus FM, Husain A (1990) Angiotensin II-forming pathways in normal and failing human hearts. Circ Res 66: 883–890. 2156635 10.1161/01.res.66.4.883

[40] Urata H, Kinoshita A, Misono KS, Bumpus FM, Husain A (1990) Identification of a highly specific chymase as the major angiotensin II-forming enzyme in the human heart. J Biol Chem 265: 22348–22357. 2266130

[41] Anderson TJ, Gerhard MD, Meredith IT, Charbonneau F, Delagrange D, et al (1995) Systemic nature of endothelial dysfunction in atherosclerosis. Am J Cardiol 75: 71B–74B. 7863979 10.1016/0002-9149(95)80017-m

[42] Panza JA, Quyyumi AA, Callahan TS, Epstein SE (1993) Effect of antihypertensive treatment on endothelium-dependent vascular relaxation in patients with essential hypertension. J Am Coll Cardiol 21: 1145–1151. 8459069 10.1016/0735-1097(93)90238-v

[43] Rossing K, Schjoedt KJ, Jensen BR, Boomsma F, Parving HH (2005) Enhanced renoprotective effects of ultrahigh doses of irbesartan in patients with type 2 diabetes and microalbuminuria. Kidney Int 68: 1190–1198. 16105050 10.1111/j.1523-1755.2005.00511.x

[44] Schmieder RE, Klingbeil AU, Fleischmann EH, Veelken R, Delles C (2005) Additional antiproteinuric effect of ultrahigh dose candesartan: a double-blind, randomized, prospective study. J Am Soc Nephrol 16: 3038–3045. 16120821 10.1681/ASN.2005020138

